# Initial Harm Reduction by N-Acetylcysteine Alleviates Cartilage Degeneration after Blunt Single-Impact Cartilage Trauma in Vivo

**DOI:** 10.3390/ijms20122916

**Published:** 2019-06-14

**Authors:** Jana Riegger, Frank Leucht, Hans-Georg Palm, Anita Ignatius, Rolf E. Brenner

**Affiliations:** 1 Division for Biochemistry of Joint and Connective Tissue Diseases, Department of Orthopedics, University of Ulm, Ulm 89081, Germany; jana.riegger@uni-ulm.de; 2 Department of Orthopedics, University of Ulm, Ulm 89081, Germany; frank.leucht@rku.de; 3 Department of Orthopedics and Trauma Surgery, German Armed Forces Hospital Ulm, Ulm 89081, Germany; hansgeorgerichpalm@bundeswehr.org; 4 Institute of Orthopedic Research and Biomechanics, University of Ulm, Ulm 89081, Germany; anita.ignatius@uni-ulm.de

**Keywords:** post-traumatic osteoarthritis, N-acetylcysteine, bone morphogenic protein 7, cartilage trauma, histomorphology, therapy

## Abstract

Joint injuries are highly associated with the development of post-traumatic osteoarthritis. Previous studies revealed cell- and matrix-protective effects of N-acetylcysteine (NAC) after ex vivo cartilage trauma, while chondroanabolic stimulation with bone morphogenetic protein 7 (BMP7) enhanced type II collagen (COL2) expression. Here, as a next step, we investigated the combined and individual efficacy of intra-articular antioxidative and chondroanabolic treatment in a rabbit in vivo cartilage trauma model. Animals were randomly divided into group A (right joint: trauma (T); left joint: T+BMP7) and group B (right joint: T+NAC; left joint: T+BMP7+NAC). Condyles were impacted with the use of a spring-loaded impact device to ensure defined, single trauma administration. After 12 weeks, histopathological analysis was performed and the presence of matrix metalloproteinase 13 (MMP-13) and COL2 was assessed. Trauma-induced hypocellularity, MMP-13 expression, and cell cluster formation were reduced in NAC-treated animals. In contrast, BMP7 further increased cluster formation. Moreover, synovial concentrations of COL2 carboxy propeptide (CPII) and proteoglycan staining intensities were enhanced in NAC- and NAC+BMP7-treated joints. For the first time, the efficacy of NAC regarding early harm reduction after blunt cartilage trauma was demonstrated in vivo. However, parallel administration of BMP7 was not significantly superior compared to NAC alone.

## 1. Introduction

The pathomechanisms that occur after knee joint injury and concurrent traumatization of the intra-articular (i.a.) cartilage are characterized by cell death, inflammation, catabolic enzyme expression, and the accumulation of reactive oxygen species (ROS), and are highly associated with the development of post-traumatic osteoarthritis (PTOA) [1,2,3]. Since the intrinsic regeneration of cartilage is very poor, early harm reduction after injury is thought to be a crucial factor in preventing PTOA.

Previously, we reported matrix- and cell-protective effects of antioxidative therapy with N-acetylcysteine (NAC) in a human ex vivo cartilage trauma model which, however, also suppressed the expression of type II collagen (COL2) [4,5]. Although chondroanabolic stimulation with bone morphogenetic protein 7 (BMP7) revealed anticatabolic and proanabolic features after ex vivo cartilage trauma, the growth factor could not countervail the impairing effects of NAC in case of parallel application [5].

For previous investigations dealing with NAC and BMP7, we used cartilage tissue of elderly patients undergoing total knee joint replacement due to osteoarthritis (OA) which might imply lower responsiveness of the chondrocytes towards anabolic stimuli, even though efficacy of BMP7 was described as being largely unaffected by age or OA [6]. Moreover, our ex vivo model does not account for all pathophysiological aspects involved in in vivo OA, nor does it account for synovial inflammation or subsequent biomechanical loading, all of which are crucial limitations. Previous studies testing NAC as therapeutic in OA animal models have not considered the specificity of a single-impact injury and associated PTOA pathogenesis [7,8]. Therefore, the present study’s aim is to clarify the remaining questions related to antioxidative and chondroanabolic treatment, both alone and in a combined manner in a rabbit in vivo cartilage trauma model, using a spring-loaded impact device for the administration of a biomechanically defined single-impact trauma [9,10].

The efficacy of the pharmacologic intervention strategies were evaluated by a well-defined rating scale, based on the Osteoarthritis Research Society International (OARSI) recommendations for pathohistological assessments of OA in rabbit models, considering the proteoglycan content and surface integrity, as well as cell density and its distribution [11]. Moreover, expression of matrix metalloproteinase 13 (MMP-13) and COL2, which are considered as representative catabolic and anabolic biomarkers in OA, were assessed in the synovial fluid as well as in the cartilage tissue.

## 2. Results

**Figure 1:**
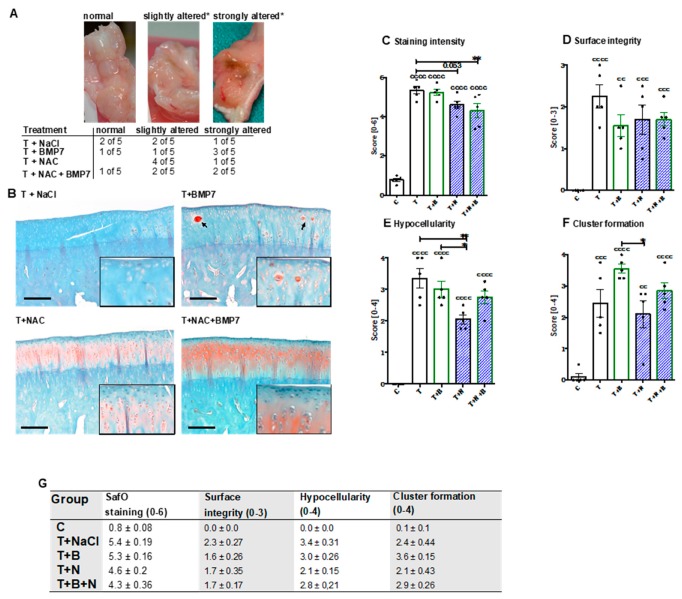
Macroscopic and histopathologic assessment of the joint capsule and SafO-stained condyle sections, respectively. (**A**)*Alteration was defined as reddish/russet color, which was considered as possible indication of inflammatory processes or intra-articular bleeding (blood residues), and vascularization. (**B**) Exemplary images of SafO-stained medial condyles of each group. Cell clusters are indicated by black arrows. The black bar represents 100 µm. (**C**–**F**) Corresponding statistical analysis of the single parameters of the histopathological assessment, charted as scattered plots with bars: (**C**) proteoglycan staining intensity, (**D**) surface integrity, (**E**) hypocellularity, and (**F**) cluster formation. (**G**) Values of the single criteria given as mean ± SEM; *n* = 5. Statistically significant differences between groups were depicted as: [vs C] cc: *p* < 0.01, ccc: *p* < 0.001, and cccc: *p* < 0.0001; [vs. T] *: *p* < 0.05, and **: *p* < 0.01. T = trauma, T+B = traumatized and BMP7-treated, T+N = traumatized and NAC-treated, T+N+B = traumatized and BMP7- plus NAC-treated.

After dissection of the joints, no macroscopic changes of the cartilage surface could be observed. The joint capsule of some animals, however, was considerably altered, in particular after BMP7 treatment (Figure 1A).

The histopathological assessment of SafO-stained sections demonstrated significant enhancement of all considered parameters after the single-impact trauma of the condyles (Figure 1C–G). Proteoglycan content was significantly preserved in NAC-treated animals with and without addition of BMP7 as indicated by higher staining intensities ([vs. T] T+N: −0.8 points, p = 0.053; T+B+N: −1.1 points; p = 0.0065) (Figure 1B,C). Enhanced staining intensities of SafO could also be found in and around cell clusters, especially after BMP7 treatment (Figure 1B). Moreover, we found moderate improvement of the surface integrity (Figure 1D) in NAC- and/or BMP7-treated animals ([vs. T] T+B: −0.7 points, T+N/T+B+N: −0.5 points). In NAC-treated animals, chondrocyte cell death (= hypocellularity, Figure 1E) was significantly lower as compared to untreated (−1.3 points; p = 0.003) or BMP7-treated (−0.95 points; p = 0.042) animals, though parallel application of BMP7 decreased the cell-protective effects of the antioxidant to some extent. Administration of BMP7 enhanced the trauma-associated incidence of cluster formation by 1.1 points (Figure 1F), causing the score to be significantly higher than that observed after NAC treatment ([vs T+N] +1.45 points; p = 0.0196). In combination with NAC, the BMP7-mediated increase of cell clusters was largely attenuated closer to that observed in the trauma-only group.

**Figure 2:**
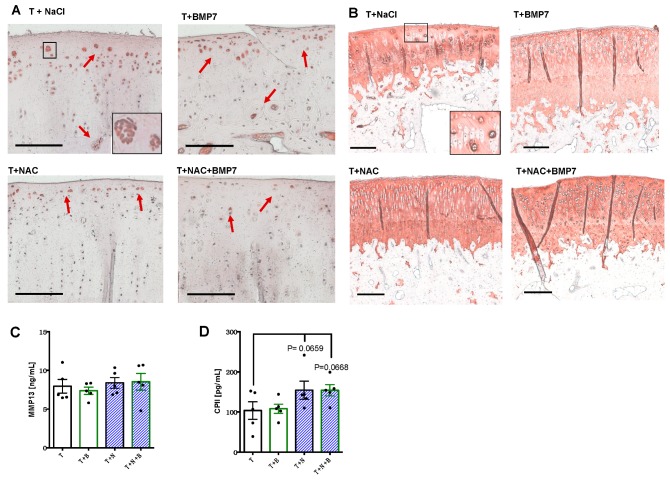
Determination of anabolic and catabolic biomarkers in cartilage and synovial fluid, respectively, after trauma with and without pharmacologic intervention. Femoral condyle sections of rabbits were immunohistochemically stained for (**A**) MMP13 and (**B**) COL2, respectively. The red arrows indicate MMP13-positive cells. The black bar represents 100 µm. Additionally, concentrations of (**C**) MMP-13 and (**D**) CPII were determined in the synovial fluid by means of specific ELISA; data values are charted as scattered plots with bars, *n* = 5. T = trauma + NaCl, T+B = traumatized and BMP7-treated, T+N = traumatized and NAC-treated, T+N+B = traumatized and BMP7- plus NAC-treated.

Immunostaining of MMP-13 revealed that positive cells were predominantly located in proximity to the surface area, the tide mark, as well as in clusters, particularly found in untreated or BMP7-treated animals (Figure 2A). In contrast, there were considerably less MMP-13-positive cartilage cells in the NAC- and NAC+BMP7-treated group. Moreover, we observed elevated COL2 staining intensities around cell clusters in all groups and a generally higher staining intensity in the NAC group with and without addition of BMP7 (Figure 2B).

In contrast to the protein detection in the cartilage tissue, quantification of MMP-13 within the synovial fluid did not reveal any difference between the groups (Figure 2C). However, absolute concentrations of CPII (Figure 2D) were noticeably enhanced from 103.7 (SEM 21.8) pg/mL in untreated joints to 154.3 (SEM 22.7) and 154.1 (SEM 14.1) pg/mL, respectively, in NAC- and NAC+BMP7-treated joints ([vs T] T+N: *p* = 0.0659; T+N+B: *p* = 0.0668).

## 3. Discussion

In accordance with our previous ex vivo studies in human cartilage explants, we found clear evidence that NAC-based antioxidative therapy possesses cell- and chondroprotective effects, as demonstrated by significant preservation of the cell count and proteoglycan content as well as decreased MMP-13 expression in chondrocytes in vivo [4,5]. Therefore, NAC might be suitable in terms of initial harm reduction after single-impact cartilage injury. In agreement with our findings, NAC has been found to prevent proteoglycan loss and reduce the pathohistological scores after i.a. fracture, as recently reported in a porcine PTOA model. These protective effects were mainly ascribed to the decrease of oxidative stress (i.e., caused by mitochondrial dysfunction) which occurs after joint trauma [12]. Moreover, NAC reduced cartilage degradation markers and exhibited comparable beneficial effects to hyaluronic acid after a single i.a. injection in patients suffering from mild to moderate knee OA [13].

Nevertheless, we previously demonstrated that BMP7 exhibited greater chondroanabolic potential after ex vivo cartilage trauma as compared to fibroblast growth factor 18 or IGF-1 [5]. Moreover, BMP7 promoted the chondrogenic phenotype, while alleviating the proinflammatory response of cartilage-derived chondrogenic stem/progenitor cells (CSPC) after stimulation with trauma-conditioned medium [14]. Overall, BMP7 was considered a promising candidate in early post-traumatic matrix regeneration, but could not be confirmed by the current results.

Although the levels of inflammatory mediators in the synovial fluid, such as IL-1b, TNF-a, and PGE2, were not determined in the present study, our previous investigations revealed a significant increase of these factors within the first week after arthrotomy and in in vivo cartilage trauma using the same model [9]. This inflammation subsided almost completely after 12 weeks, confirming that inflammation might be primarily associated with the early phase of joint injury and PTOA pathogenesis [2]. Inflammatory processes and subsequent ROS generation are known to trigger chondrocyte death as well as the expression of catabolic enzymes and direct ECM breakdown [3]. Administration of NAC might have attenuated these harmful effects due to its antioxidative characteristics, which prevented hypocellularity and facilitated possible regenerative processes as indicated by enhanced CPII levels in the synovial fluid. It is rather unlikely that NAC directly induced COL2 expression, due to its inhibitory effects on the chondrogenic phenotype and COL2 biosynthesis [4,5,14].

Although Hurting et al. reported that early intervention with BMP7 (i.a. injections at day 0 and day 7 after surgery) was suitable to prevent progression of cartilage degeneration in a comparable sheep in vivo cartilage trauma model [6], other sources claim that the chondroanabolic efficacy of BMP7 is impaired by IL-1b [15], which rather argues against immediate BMP7 administration during acute inflammation in the early phase. Overall, we suggest that later application of BMP7—and perhaps after initial harm reduction by NAC administration—might circumvent the possible inhibitory effects of the inflammatory response after surgery and cartilage injury. That NAC-mediated suppression of COL2 synthesis can be successfully restored in a sequential application regimen using BMP7 could be demonstrated during chondrogenic differentiation of CSPC [14].

Nevertheless, enhanced incidence of cluster formation in the cartilage and notable alteration of the joint capsule in BMP7-treated animals indicated promitotic and possible proinflammatory effects. In accordance with these observations, we previously found that BMP7 significantly increased the proliferation of CSPC and induced cell cluster formation in human cartilage after ex vivo trauma [5,14]. In contrast, we observed that NAC inhibited proliferation of cartilage cells [4,5], which might explain attenuating effects towards trauma- and BMP7-induced cell cluster formation in the present study. Possible synovial alteration might be based on the fact that BMPs are strongly involved in angiogenesis [16]. This process is driven by increased expression of vascular endothelial growth factor, cytokines, and chemokines, which has also been reported in a clinical study after autologous bone graft transplantation and additional BMP7 application [17].

The major limitations of our study can be ascribed to missing synovial fluid samples of nonoperated animals as well as the rather small sample size of all groups. Furthermore, the optimal dosage and way of administration of the therapeutic substances have to be determined to avoid repeated i.a. injections.

Overall, these novel results confirm the efficacy of NAC concerning initial harm reduction after blunt cartilage injury as an in vivo proof of principle and reinforce our hypothesis that a sequential rather than a parallel application of BMP7 might be beneficial in terms of cartilage regeneration.

## 4. Materials and Methods

### 4.1. Animal Model

The animal study was approved by the local legal representative (regional council Tuebingen; registration number 1112) and conducted in agreement with the European Convention for the Protection of Vertebrate Animals used for Experimental and Other Scientific Purposes. Ten female New Zealand White (NZW) rabbits (24 weeks old) were randomly divided in two groups (Table 1): group A (right joint: trauma (T), untreated; left joint: T, treated with BMP7 (1 µg; PeproTech, Hamburg, Germany)) and group B (right joint: T, treated with NAC (5 mg; Ratiopharm, Ulm, Germany); left joint: T, treated with BMP7+NAC). Therapeutic concentrations were chosen according to previous in vivo studies [7,18]. For histopathologic assessment, the medial condyles (left joint) of 5 unoperated NZW rabbits (same gender and age), which belonged to another study [9] and did not receive i.a. injection, were used as controls (C).

Animals were sedated with atropine sulfate (0.4 mL/kg) and anesthetized using an initial intravenous (i.v.) dose of ketamine hydrochloride (15 mg/kg) and xylazine hydrochloride (1 mg/kg). Anesthesia was maintained by inhalation of 2% isoflurane. While animals of group B additionally received 100 mg/kg NAC by i.v. infusion during the operation to prolong therapeutically relevant i.a. levels of the antioxidant, group A received equivalent volumes of 0.9% NaCl.

**Figure 3:**
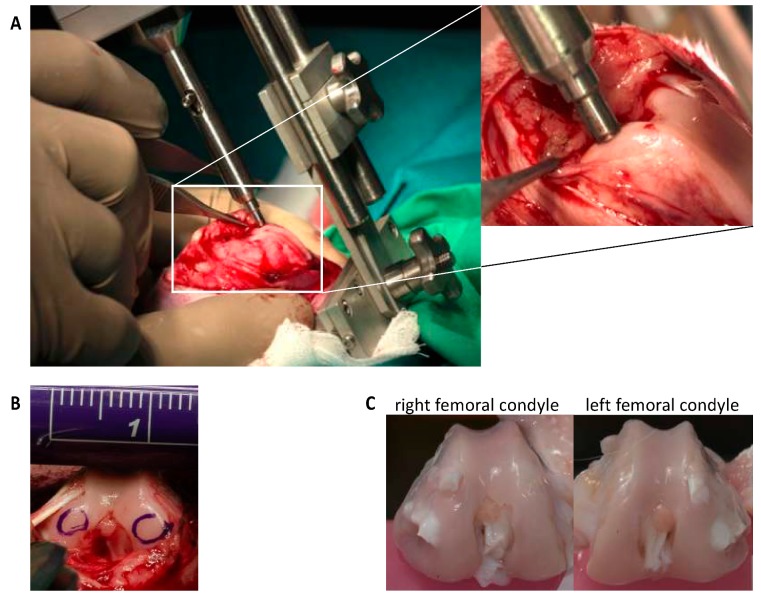
The rabbit in vivo cartilage trauma model. (**A**) The spring-loaded impact device for biomechanically defined force administration is fixed to the femur to avoid a shift of the indenter tip during the impact. (**B**) Both medial and lateral condyles were impacted. The impact sites are demonstrated by encirclement. (**C**) After 12 weeks, the animals were euthanized and the femoral condyles were harvested.

After arthrotomy, impaction of both femoral condyles was performed by means of a specially designed spring-loaded impact device (Figure 3), with an energy of 1.0 J as previously described [9,10]. Afterwards, NAC and BMP7 were intraoperatively applied by i.a. injection with a total volume of 400 µL (= first injection). The same volume of 0.9% NaCl served as sham in untreated joints. A second injection was administered 48h postsurgery under short i.v. anesthesia (described above). Postsurgical pain management included buprenorphine subcutaneous injection (0.03 mg/kg; intraoperative, 6 and 18 h after surgery) and oral tramadol administration via drinking water (25 mg/mL; 3 days before/after surgery). The rabbits received subcutaneous enrofloxacin (7.5 mg/kg for 3 days) as antibiotic treatment and regular clinical wound controls were performed (additional information: supplementary material S1). Rabbits were euthanized by an overdose of thiopental 12 weeks after surgery.

### 4.2. Histopathological Assessment

Both femoral condyles were fixed (4% paraformaldehyde), decalcified (20% EDTA, pH 7.4), and embedded in paraffin. Proteoglycan staining by means of safranin-O (SafO) allowed for histopathological assessment. In short, dewaxed and rehydrated sections were stained with SafO (Fisher Scientific, Schwerte, Deutschland) and Fast Green (Sigma-Aldrich, Taufkirchen, Germany), followed by a final staining of the cell nuclei by Gill’s haematoxylin No. 3 (Sigma-Aldrich) and documentation with an Axioskop 2 mot plus (Zeiss, Oberkochen, Germany). The corresponding scoring criteria and their respective specifications, which were principally derived from Laverty et al. [11], are depicted in the supplementary material S2. The scoring was performed independently by two observers (interclass correlation: 0.81).

### 4.3. Immunohistochemistry

Specific antibodies were used to analyze the presence of COL2 and MMP-13 (both: Acris, Hiddenhausen, Germany) in cartilage. Dewaxed and rehydrated sections of the medial condyle were predigested for 30 min at 37 °C for antigen retrieval with pepsin (1 mg/mL in 0.5 M acetic acid) in the case of COL2, or incubated in citrate buffer (pH 6.0) at 60 °C overnight in the case of MMP-13. The staining was performed with the Dako LSAB2 System-HRP kit (Dako, Glostrup, Denmark). Subsequent staining of the cell nuclei and documentation were performed as described above.

### 4.4. Quantification of MMP-13 and CPII in the Synovial Fluid

Synovial fluid was aspirated from the joint cavity after i.a. injection of 1 mL PBS (PAA Laboratories, Cölbe, Germany) and repeated flexion of the joint. The concentration of secreted MMP-13 (catabolic marker) was determined using a rabbit MMP13 ELISA Kit (MyBioSource, San Diego, California, USA). Evaluation of COL2 synthesis (anabolic marker) was performed using a rabbit CPII ELISA (MyBioSource), which binds type II collagen carboxy propeptide (CP II) cleaved from procollagen II after its release into the matrix.

### 4.5. Statistical Analysis

Statistical analysis was performed by using GraphPad Prism version 6.0h (GraphPad Software, San Diego, CA, USA). A one-way analysis of variance (ANOVA) with subsequent Bonferroni multiple comparison post-test was applied on the data sets, in which a *p*-value lower than 0.05 was considered significant.

## Figures and Tables

**Table 1:** Grouping and corresponding treatment scheme of the in vivo experiment.

	Animal Group	
	A (*n* = 5)	B (*n* = 5)
Right knee	Trauma (T) + 0.9% NaCl	T + NAC
Left knee	T + BMP7	T + NAC + BMP7

## Supplementary Materials

Supplementary Materials can be found at https://www.mdpi.com/1422-0067/20/12/2916/s1.

## Abbreviations

ANOVA	One-way analysis of variance
BMP7	Bone morphogenetic protein 7
C	Control
COL2	Type II collagen
CPII	Type II collagen carboxy propeptide
CSPC	Chondrogenic stem/progenitor cells
MMP-13	Matrix metalloproteinase 13
NAC	N-acetylcysteine
NZW	New Zealand White rabbits
PTOA	Post-traumatic osteoarthritis
ROS	Reactive oxygen species
SafO	Safranin-O
T	Trauma
