# Supplementary material for: Initial Harm Reduction by N-Acetylcysteine Alleviates Cartilage Degeneration after Blunt Single-Impact Cartilage Trauma in Vivo
Source: Int J Mol Sci. 2019 Jun 14;20(12):2916. doi: 10.3390/ijms20122916 (PMC6628290; doi:10.3390/ijms20122916)
Supplement: Supplementary file 1 [file ijms-20-02916-s001.pdf]

S1 Further information about the health status and housing of the animals

Rabbits were hold in double cages (2 animals each) according to the official European Guidelines 2010/63/EU. Directly after the operation, animals were separated until wounds were completely closed.

Hygiene status was monitored according to the official FELASA criteria. The weight monitoring as well as the daily wound and behavior controls did not show alarming anomalies. However, uncomplicated hematoma formation around wounds of animals receiving NAC seemed to be more pronounced as compared to animals which did not receive NAC.

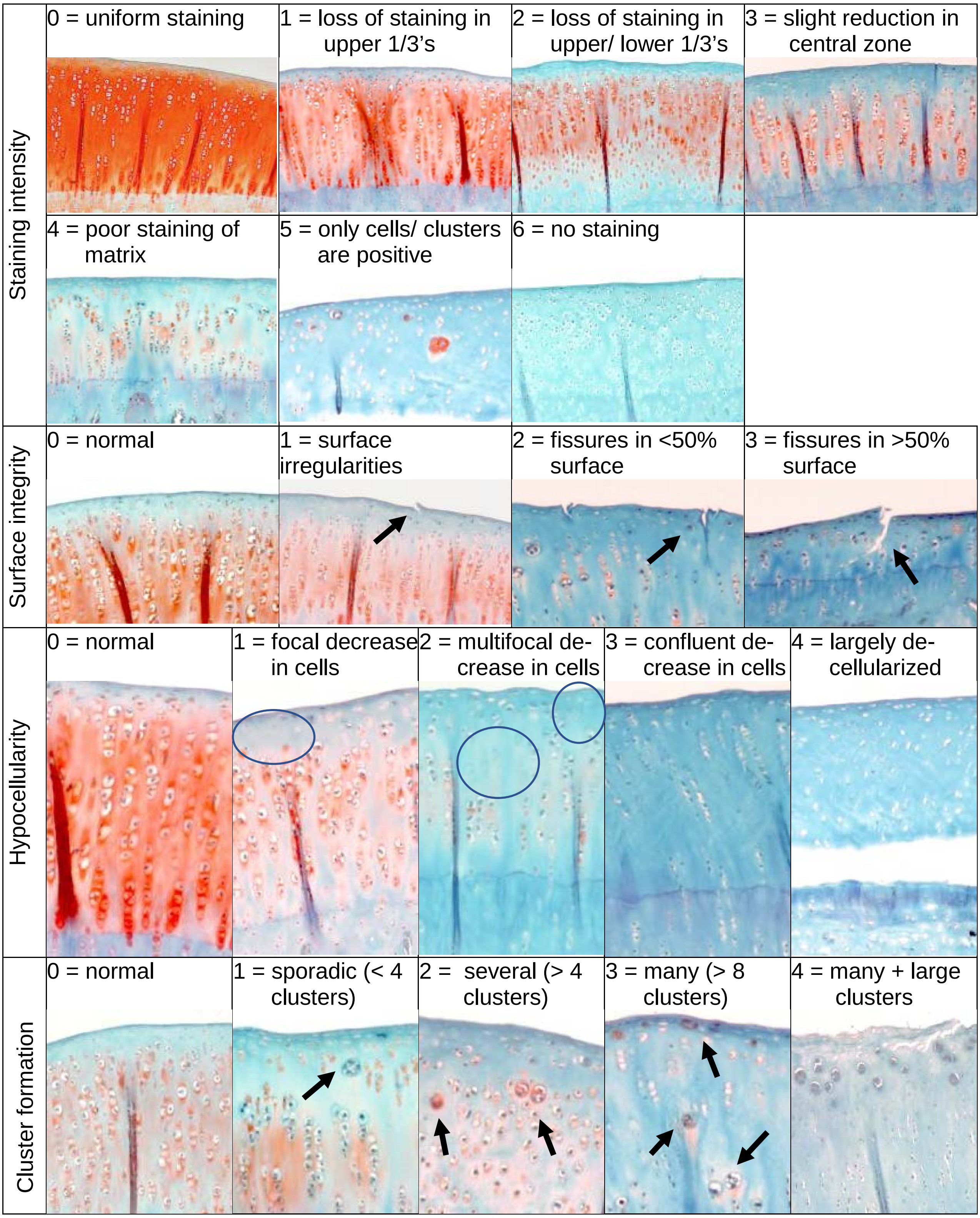

Figure S2: Scoring criteria and respective specifications. Semiquantitative assessment of safranin-O stained cartilage sections was performed by using the criteria “staining intensity” (ranging from 0 to 6 points), “surface integrity” (ranging from 0 to 3 points) as well as “hypocellularity” and “cluster formation” (both ranging from 0 to 4 points).
